# Editorial: Implementation science to address health disparities and improve the equitable implementation of proven interventions

**DOI:** 10.3389/fpubh.2023.1356063

**Published:** 2024-01-11

**Authors:** Karla I. Galaviz, Mechelle Sanders, Oscar Gil

**Affiliations:** ^1^Department of Applied Health Science, Indiana University School of Public Health-Bloomington, Bloomington, IN, United States; ^2^Department of Family Medicine, University of Rochester, Rochester, NY, United States; ^3^Department of Anthropology, University at Buffalo, Buffalo, NY, United States

**Keywords:** health disparities, knowledge translation, implementation research, dissemination and implementation, health equity (MeSH)

The constitution of the WHO states that “every human being should enjoy the highest attainable standard of health without distinction of race, religion, political belief, economic or social condition.” Yet, this fundamental right is not fully enjoyed by all people, as decades of health disparities research have documented. Health disparities are fueled by social and structural determinants of health, including racism and discrimination (1), which shape access, implementation, and sustainability of health interventions or services. Implementation science offers perspectives and tools that can be harnessed to promote health equity (2–4), and several recommendations on how to achieve this have been published (5–10).

Experts state that the implementation science field has fallen short in terms of designing equitable implementation strategies, employing equity-focused metrics, integrating equity in frameworks, and working with organizations/sectors outside healthcare (2, 3). The studies published in this Research Topic tackle these shortcomings by employing meaningful engagement and partnerships with community members and organizations, identifying implementation determinants unique to underserved settings and strategies to address them, and introducing tools to co-producing health interventions and assessing bias in healthcare decision making. In addition, new ideas on how to address equity in sustainability efforts and how to re-shape the implementation science field are introduced which open research areas for implementation scientists to pursue. These studies are authored by a range of implementation scientists, including well-established leaders in the field and early career implementation scientists from under-represented groups.

Five studies in this Research Topic identified implementation determinants which are factors believed or empirically shown to influence implementation outcomes (11). González-Casanova et al. identified implementation determinants for mental health promotion practices among *promotores* serving immigrants in Mexican consulates in the United States. Seth et al. identified organizational factors associated with the adoption of pre-exposure prophylaxis therapy among family planning clinics in the Southern United States. Singh et al. examined barriers and facilitators to providing, accessing, and receiving LGBTQ+ affirming care within the Veteran's Health Administration among clinicians and veterans. Fuster et al. examined the outcomes and implementation determinants of interventions co-developed using Human-Centered Design in two Latin American restaurants in New York. Finally, Itanyi et al. identified implementation determinants of cervical cancer control practices in the existing HIV care infrastructure in Nigeria and strategies to address them. To avoid inadvertently reinforcing health inequities (12), implementation strategies should address the context-specific determinants present in minority-serving settings. These studies identify several determinants that can be addressed through equity-promoting implementation strategies.

Three studies in this Research Topic focused on developing community partnerships to promote the equitable implementation of health interventions in the United States. Akintobi et al. described an evaluation of the Community Engaged Course and Action Network developed in the state of Georgia. Authors provide lessons to strengthen community-based participatory research principles and partnerships to improve health outcomes among communities of color. Blebu et al. described how cross-sector partnerships helped identify implementation factors related to racial disparities in adverse birth outcomes among marginalized populations in California. Finally, Steinman et al. described how partnerships with community-based organizations helped identify implementation strategies to improve access to depression care among underserved older adults in Washington and California. As previously recommended (12), these studies prioritize the needs of community partners and describe how implementation science can foster community resilience and active engagement.

Developing the science of adaptation has been recommended to advance health equity in implementation science (3) and two studies in this Research Topic address this area. Hess and Davis adapted the *Community Guide* recommendations for increasing physical activity in rural community settings and demonstrated adaptation and context relevance were critical to the dissemination of recommendations in rural communities. Woodard et al. adapted a suicide safety planning intervention using peer support in rural areas and provide a comprehensive assessment of barriers and facilitators to implementing an adapted version of the model. These studies provide useful examples of systematic processes for conducting planned adaptations; their findings highlight the importance of improving the fit and relevance of health interventions for rural communities.

Two other studies in this Research Topic introduced new research tools to promote health equity. Yardley et al. introduced the Agile Co-production and Evaluation framework for developing public health interventions, messaging, and guidance. The framework seeks to inform efforts to rapidly develop interventions and messaging by combining co-production methods with large-scale testing and real-world evaluation. Pool et al. introduced a tool to assess bias during team-based clinical decision-making. The tool can be used to promote a more equitable decision-making processes in healthcare by identifying the presence of team-based bias, promoting reflexivity, and informing implementation strategy design and testing. Future use of these tools will determine their utility and potential to promote health equity.

Finally, two perspectives in this Research Topic introduced new areas the implementation science field should pursue to promote health equity. The first perspective from Shelton et al. discussed how a health equity framing is essential to sustaining evidence-based interventions in under-resourced communities. This perspective focuses on identifying and nurturing existing assets within individuals and communities and provides recommendations to make progress toward sustainability. A perspective by Bradley et al. introduced a conceptual frame for integrating scholarship from the Black Radical Tradition in implementation science. Through a disciplinary self-critique of the field, authors call for a re-alignment of implementation science to focus on examining and dismantling systems that perpetuate racial inequalities. These perspectives open opportunities to explore novel equity-related issues in the implementation science field.

The studies published in this Research Topic offer several equity-focused lessons for the implementation science field and identify future directions to pursue (Figure 1). First, implementation science should be reframed from a “rubric of scarcity” to one that fosters the resilience of historically underserved communities who are engaged as active partners (13). Implementation research efforts should thus follow community-centered approaches that foster resilience among minoritized communities and promote active engagement through shared power and decision making. Second, to avoid inadvertently reinforcing health inequities (12), implementation science should prioritize the history of struggle among minoritized populations to gain access to health. Any efforts to reduce health disparities should be designed and implemented through the lens of this historical struggle. Finally, implementation science should focus on addressing the structural systems that perpetuate health inequities. A structural competency framework (14) could be adopted to dismantle the systems that lead to poor access to and implementation of evidence-based interventions.

**Figure 1:**
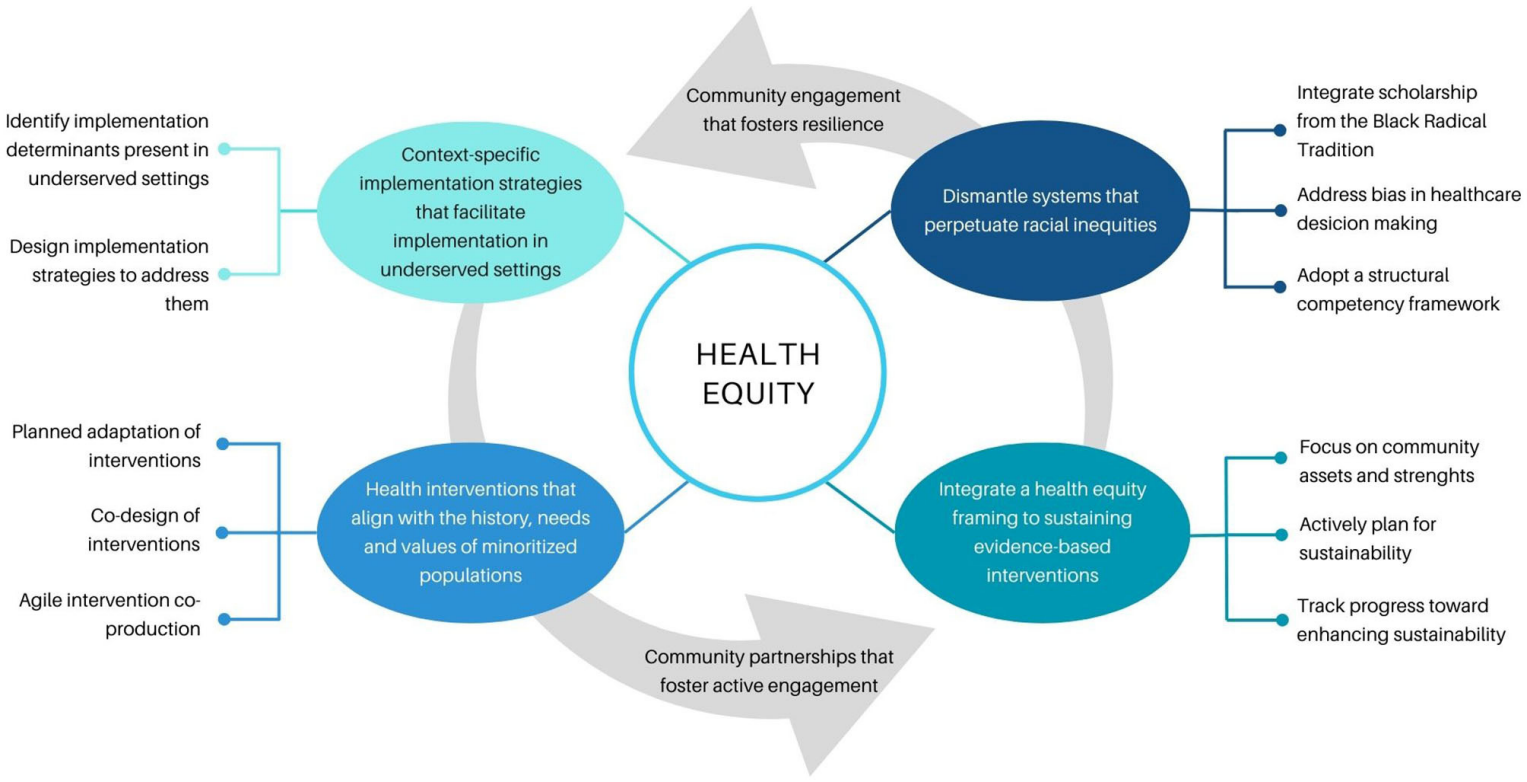
Contributions of the studies published in this Research Topic.

In closing, substantial work is needed to ensure every person enjoys the highest attainable standard of health. This Research Topic offers ideas to capitalize on the potential the implementation science field has to promote good health for all.

## Author contributions

KG: Conceptualization, Writing – original draft, Writing – review & editing. MS: Writing – review & editing. OG: Writing – review & editing.

## References

[1] Jones NL Breen N Das R Farhat T Palmer R. Cross-cutting themes to advance the science of minority health and health disparities. Am J Pub Health. (2019) 109:S21–s4. 10.2105/AJPH.2019.304950 30699031 PMC6356138

[2] Brownson RC Kumanyika SK Kreuter MW Haire-Joshu D. Implementation science should give higher priority to health equity. Implem Sci. (2021) 16:28. 10.1186/s13012-021-01097-0 33740999 PMC7977499

[3] Baumann AA Cabassa LJ. Reframing implementation science to address inequities in healthcare delivery. BMC Health Serv Res. (2020) 20:190. 10.1186/s12913-020-4975-3 32164706 PMC7069050

[4] Galaviz KI Breland JY Sanders M Breathett K Cerezo A Gil O. Implementation science to address health disparities during the coronavirus pandemic. Health Equity. (2020) 4:463–7. 10.1089/heq.2020.0044 33111032 PMC7585610

[5] Shelton RC Chambers DA Glasgow RE. An extension of RE-AIM to enhance sustainability: addressing dynamic context and promoting health equity over time. Front Pub Health. (2020) 8:134. 10.3389/fpubh.2020.00134 32478025 PMC7235159

[6] Woodward EN Matthieu MM Uchendu US Rogal S Kirchner JE. The health equity implementation framework: proposal and preliminary study of hepatitis C virus treatment. Implem Sci. (2019) 14:26. 10.1186/s13012-019-0861-y 30866982 PMC6417278

[7] Eslava-Schmalbach J Garzón-Orjuela N Elias V Reveiz L Tran N Langlois EV. Conceptual framework of equity-focused implementation research for health programs (EquIR). Int J Equity Health. (2019) 18:80. 10.1186/s12939-019-0984-4 31151452 PMC6544990

[8] Shelton RC Adsul P Oh A. Recommendations for addressing structural racism in implementation science: a call to the field. Ethn Dis. (2021) 31:357–64. 10.18865/ed.31.S1.357 34045837 PMC8143847

[9] Kerkhoff AD Farrand E Marquez C Cattamanchi A Handley MA. Addressing health disparities through implementation science—a need to integrate an equity lens from the outset. Implem Sci. (2022) 17:13. 10.1186/s13012-022-01189-5 35101088 PMC8802460

[10] Shelton RC Adsul P Oh A Moise N Griffith DM. Application of an antiracism lens in the field of implementation science (IS): recommendations for reframing implementation research with a focus on justice and racial equity. Implem Res Prac. (2021) 2:26334895211049482. 10.1177/26334895211049482 37089985 PMC9978668

[11] Nilsen P Bernhardsson S. Context matters in implementation science: a scoping review of determinant frameworks that describe contextual determinants for implementation outcomes. BMC Health Serv Res. (2019) 19:189. 10.1186/s12913-019-4015-3 30909897 PMC6432749

[12] Beidas RS Dorsey S Lewis CC Lyon AR Powell BJ Purtle J. Promises and pitfalls in implementation science from the perspective of US-based researchers: learning from a pre-mortem. Implem Sci. (2022) 17:55. 10.1186/s13012-022-01226-3 35964095 PMC9375077

[13] Keshavjee S. Justifying a Lower Standard of Health Care for the World's Poor: A Call for Decolonizing Global Health. In:Biehl J Adams V, editors. Arc of Interference: Medical Anthropology for Worlds on Edge, Critical Global Health: Evidence, Efficacy, Ethnography. Durham: Duke University Press (2023). p. 91–111.

[14] Hester RJ. Embodied Politics Indigenous Migrant Activism, Cultural Competency, and Health Promotion in California. New Brunswick, NJ: Rutgers University Press (2022).

